# Case Report: Androgen receptor-positive, locoregionally advanced breast cancer in a transgender man and an update on breast cancer management in a gender-diverse patient population

**DOI:** 10.3389/fonc.2025.1654048

**Published:** 2025-09-25

**Authors:** Yastira Ramdas, Trenton Reinicke, Carolyn A. Schnurr, Laura Nadeau, Dana Zakalik, Sharon W. Lahiri, Andrea Brudvik, Sayee Kiran, Christina M. Busuito, Joshua T. Dilworth

**Affiliations:** ^1^ Department of Radiation Oncology, Corewell Health William Beaumont University Hospital, Royal Oak, MI, United States; ^2^ Department of Radiation Oncology, Oakland University William Beaumont School of Medicine, Rochester, MI, United States; ^3^ Department of Breast Surgery, Corewell Health William Beaumont University Hospital, Royal Oak, MI, United States; ^4^ Department of Medical Oncology, Corewell Health William Beaumont University Hospital, Royal Oak, MI, United States; ^5^ Department of Medical Oncology and Cancer Genetics, Corewell Health William Beaumont University Hospital, Royal Oak, MI, United States; ^6^ Department of Endocrinology, Henry Ford Health System, Detroit, MI, United States; ^7^ Department of Radiology, Corewell Health William Beaumont University Hospital, Royal Oak, MI, United States; ^8^ Department of Plastic Surgery, Somerset Plastic Surgery, Troy, MI, United States

**Keywords:** male breast cancer, transgender, androgen receptor, invasive lobular carcinoma, testosterone therapy, personalized treatment

## Abstract

The role of androgen receptor (AR) signaling in breast cancer is underexplored and may be particularly important in the treatment of patients with higher levels of circulating androgens. We discuss the management of a 70-year-old, postmenopausal transgender man with a six-and-one-half-year history of testosterone therapy, who presented with locoregionally advanced, invasive lobular carcinoma with apocrine features that was estrogen receptor (ER)-negative, progesterone receptor (PR)-negative, human epidermal growth factor receptor 2 (HER2)-positive, and AR-positive. The approach to discontinue his testosterone indefinitely upon diagnosis was determined through shared-decision making with the patient. He received neoadjuvant HER2-directed chemotherapy and achieved a complete metabolic response. He underwent bilateral total mastectomies with left targeted axillary lymph node dissection. Final pathology showed a near complete pathologic response in the breast and a pathologic complete response in three sentinel lymph nodes. He completed a course of conventionally fractionated left chest wall and regional nodal proton beam irradiation and received adjuvant HER-2 directed therapy. He tolerated treatment well and remains disease-free two years since diagnosis. This case report and review underscore the importance of a multi-disciplinary and nuanced approach to personalized management of breast cancer in a gender-diverse patient population. Continued characterization of the AR as a potential therapeutic target in patients with breast cancer is warranted

## Introduction

The majority (70-80%) of breast cancer is estrogen receptor (ER)-positive, and blocking the ER with endocrine therapy is a cornerstone of the treatment of all stages of ER-positive disease (1). The androgen receptor (AR) is also expressed in the majority (~75%) of all breast cancer with variable expression among biological subtypes (2–6). The impact that AR expression has on breast cancer progression depends on the co-expression or absence of ER and human epidermal growth factor receptor 2 (HER2) and on the balance of circulating estrogens and androgens (7–15). With relatively limited clinical data characterizing the therapeutic benefit of systemic agents that either inhibit or potentiate AR signaling, AR expression is not routinely determined (16).

AR expression may be particularly relevant for patients with high levels of circulating androgens (either because of high endogenous production or receipt of androgen therapy). Cisgender men and transgender men with breast cancer constitute an underrepresented and understudied patient population, yet these patients may derive considerable benefit from therapies that modulate the AR. Here we discuss the management of a 70-year-old postmenopausal, transgender man who presented with ER/progesterone receptor (PR)-negative, HER2-positive, AR-positive locoregionally advanced breast cancer. We provide a review of the AR as an emerging therapeutic target in breast cancer and an update of breast cancer management in the transgender patient population.

## Case description

Our patient provided written, informed consent for this case report. He self-identifies as a Non-Hispanic White, transgender man with pronouns he/him/his. He underwent menopause at age 52 and received routine cervical cancer screening until age 50 and routine screening mammography until presentation at age 70. At age 64, he began weekly testosterone injections (50 mg) as part of his gender-affirming hormone therapy, and his serum total testosterone measured 709 ng/dL three months prior to his diagnosis of breast cancer.

**Figure 1:**
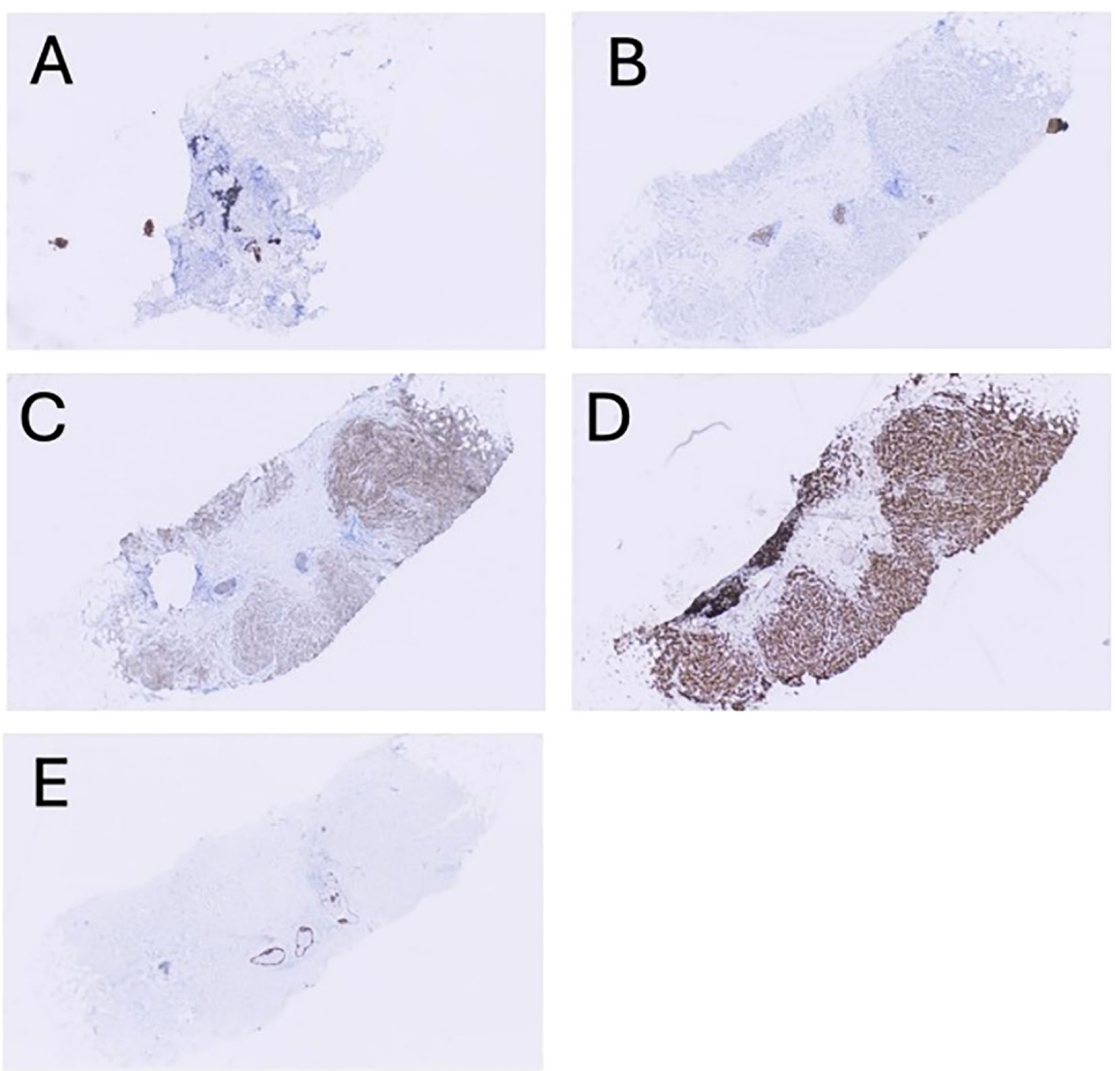
Representative, low-magnification (4x) images showing immunohistochemical staining results for hormonal and adhesion markers: **(A)** ER (estrogen receptor), **(B)** PR (progesterone receptor), **(C)** HER2 (human epidermal growth factor receptor 2), **(D)** AR (androgen receptor), and **(E)** E-cadherin (ECAD).

**Figure 2:**
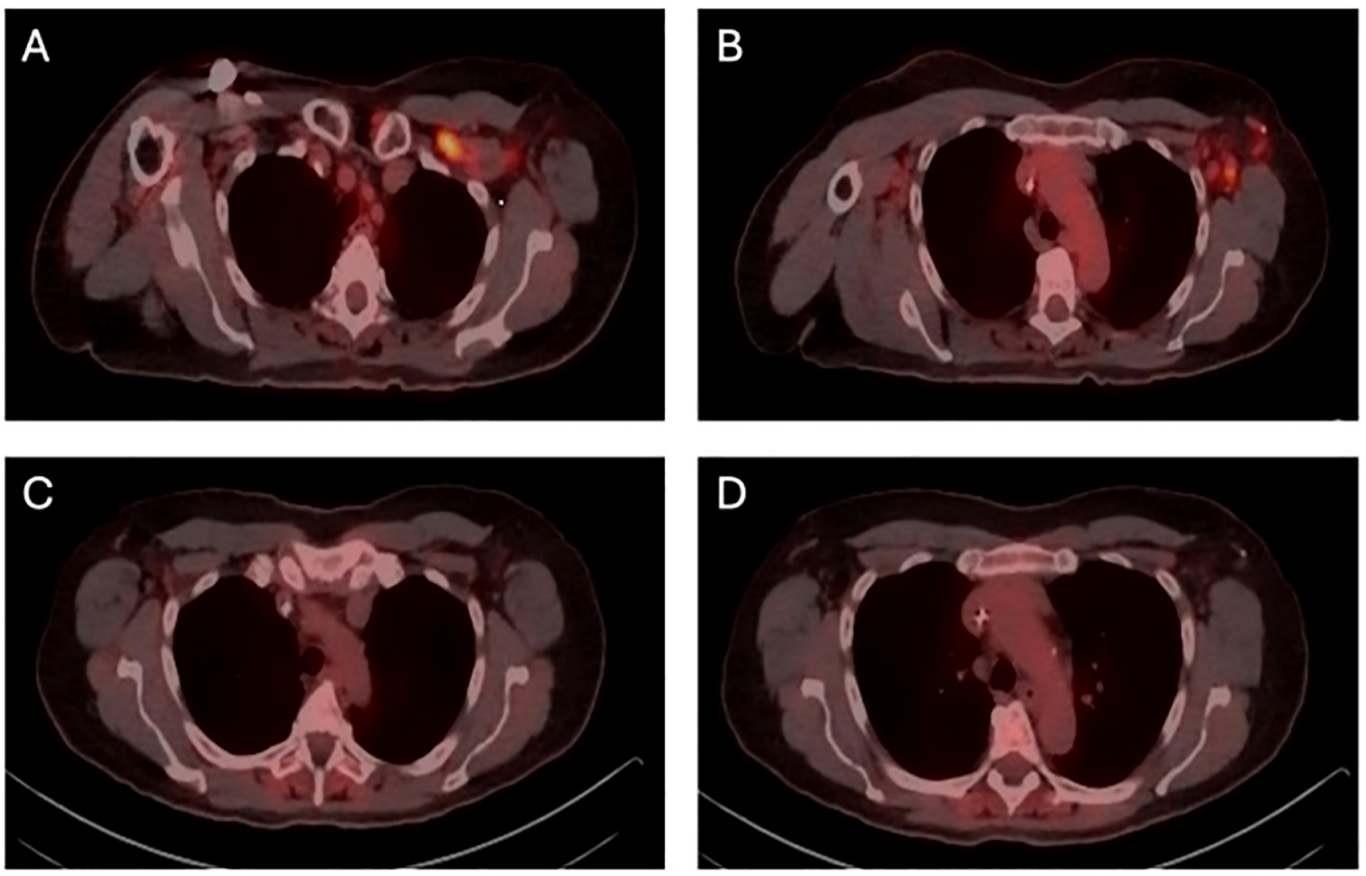
FDG-PET (fluorodeoxyglucose-positron emission tomography) demonstrating metabolic response to systemic therapy. **(A)** initial FDG-PET prior to neoadjuvant chemotherapy, showing uptake in superior axillary (levels I, II, and III) lymph nodes **(B)** initial FDG-PET prior to neoadjuvant chemotherapy, showing uptake in inferior axillary (level I and II) lymph nodes. **(C)** re-staging FDG-PET following neoadjuvant chemotherapy, showing metabolic complete response in superior axillary lymph nodes. **(D)** re-staging FDG-PET following neoadjuvant chemotherapy, showing metabolic complete response in inferior axillary (levels I and II) lymph nodes.

The patient presented in September 2023 with pain and “swelling” in his left underarm. Diagnostic imaging identified a 2.7 cm mass in the central portion of his left breast and abnormal-appearing lymph nodes in the ipsilateral axilla. Review of the core needle biopsy of the breast and an axillary lymph node confirmed grade 2 invasive lobular carcinoma with apocrine features. The carcinoma was ER-negative (0% staining), PR-negative (0% staining), HER2-positive (2+ staining by immunohistochemistry and positive on *in situ* hybridization with a ratio of 2.70), and AR-positive (diffuse, strong staining) (**Figure 1**). Fluorodeoxyglucose-positron emission tomography (FDG-PET) revealed glucose uptake in the central left breast mass and in multiple ipsilateral axillary (levels I, II, and III) lymph nodes but no distant metastasis (**Figure 2**). The patient was assigned with anatomic/clinical prognostic stage IIIC/IIIB (cT2, cN3a(f), cM0) disease. Germline genetic testing did not reveal any pathogenic or likely pathogenic variants.

The patient received neoadjuvant chemotherapy with 6 cycles of docetaxel, carboplatin, trastuzumab, and pertuzumab. FDG-PET following neoadjuvant therapy supported a complete metabolic response. He underwent bilateral total mastectomies with a targeted axillary dissection on the left. Pathology revealed a 2.5 mm residual focus of grade 3 invasive lobular carcinoma in the left breast. All surgical margins were clear by at least 2 mm. There was a complete pathologic response in three recovered axillary lymph nodes, including the biopsied lymph node. He completed a course of conventionally fractionated, intensity modulated proton beam irradiation directed to the left chest wall and axillary (levels I, II, and III), internal mammary, supraclavicular, and posterior cervical lymph nodes. He received adjuvant trastuzumab emtansine.

The conversation surrounding the patient’s gender-affirming hormone therapy was compassionate, nuanced, and non-dogmatic. The potential oncological risk of continued testosterone therapy was weighed against the profound impact that withdrawal of this treatment might have on the patient’s gender identity and mental health. Ultimately, the treatment approach was determined through shared decision-making with the patient. A dialogue between the patient, his cancer treatment providers (including his breast surgeon, medical oncologist, and radiation oncologist), his endocrinologist (also the prescriber of his testosterone), and his counselor was initiated at the time of diagnosis, maintained throughout his cancer treatment, and continues into his survivorship care.

It was explained to the patient that there was a potential role of continued high levels of circulating androgens in promoting the growth of androgen receptor-positive cancer cells, which may increase the risk of cancer recurrence. While the clinical benefits of estrogen blockade in cisgender women with ER-positive breast cancer and of androgen blockade in select cisgender men with prostate cancer are well established, we explained to the patient that the role of the AR in cisgender women with breast cancer is still unclear—and that the role of the AR in transgender men taking testosterone is even less well studied. The consensus recommendation from his treating physicians and from our institution’s multi-disciplinary breast tumor board was for him to consider discontinuing his testosterone therapy. The patient was asked where he was on the spectrum between being strongly opposed and strongly motivated to discontinue his testosterone. We discussed his feelings toward discontinuing this treatment indefinitely, resuming the treatment after a certain period of cancer-free survival, continuing on a reduced dose of testosterone, and continuing on his current dosage. Ultimately, the patient decided that “any risk” that testosterone therapy might have on increasing cancer recurrence made it worth seeing how he would tolerate stopping this treatment.

**Figure 3:**
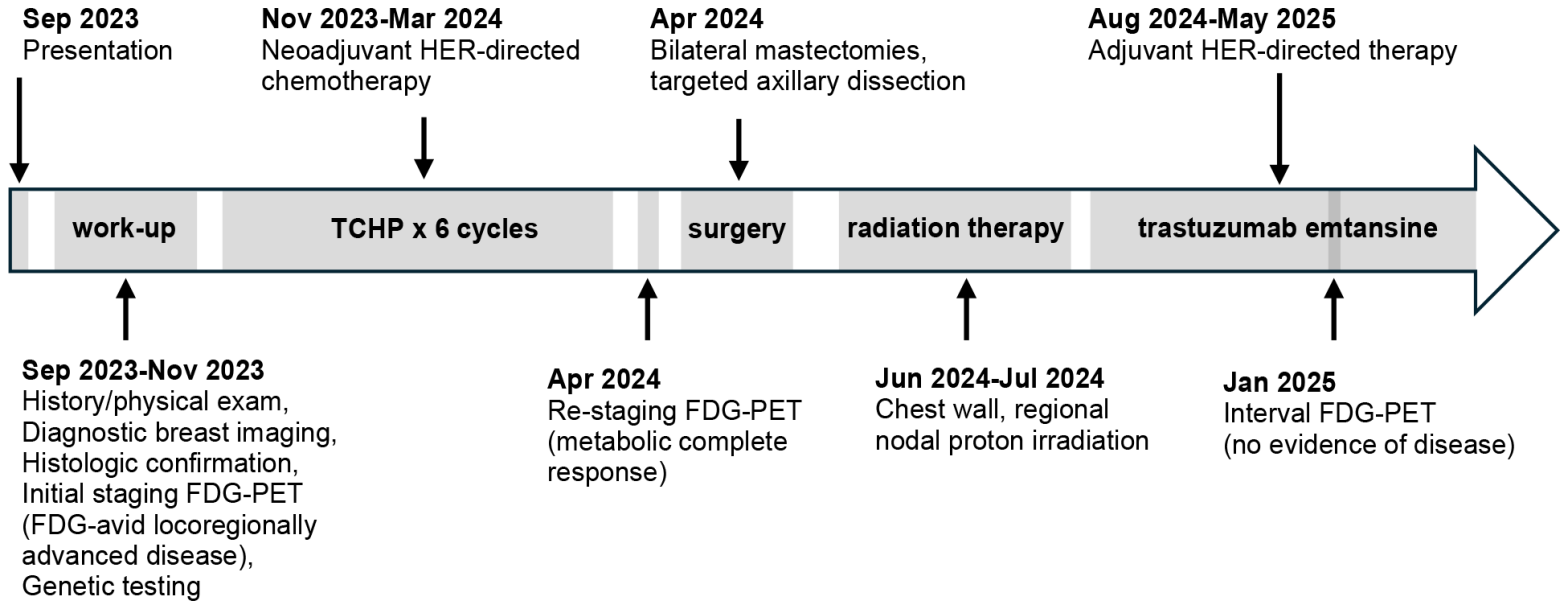
Treatment schema.

With discontinuation of testosterone, he experienced decreased energy, arthralgias, and insomnia. He also experienced changes that worsened his gender dysphoria, including redistribution of fat to his face, hips, and buttocks, decreased density of his beard, and increase pitch of his voice. At the patient’s last follow-up nearly two years after diagnosis, the patient stated he still “misses his thick beard, his energy, and his narrow hips” but that he remains motivated to not re-initiate his testosterone therapy. He manages his symptoms with diet, exercise (biking, swimming, and walking), meditation, and regular visits with his counselor. He remains disease free at the time of this publication. **Figure 3** shows the patient’s treatment timeline.

## Discussion

### Epidemiology

Breast cancer is the most common non-cutaneous cancer diagnosed in cisgender women, affecting approximately 1 in 8 (~143 cases per 100,000 person years) (17). Breast cancer in cisgender men accounts for less than 1% of all breast cancer diagnoses and affects approximately 1 in 833 (~1 case per 100,000 person years) (17). Transgender individuals carry an intermediate risk of breast cancer. One study reported a collective ~43 cases per 100,000 person years for a cohort of 2,260 transgender women and 1,229 transgender men (18). The rate of breast cancer in the transgender population, however, is highly variable and dependent upon the type and duration of gender-affirming hormone therapy these individuals receive (19–22). Estrogens promote development of mammary tissue in transgender women. These changes, including the formation of ducts, lobules, and acini, may become histologically and radiographically indistinguishable from cisgender women (23–26). Receipt of feminizing hormones also leads to a higher frequency of benign breast processes (such as cysts and fibroadenomas) and malignant transformation of breast tissue. Conversely, testosterone causes atrophy of breast tissue in transgender men, which may lower the risk of malignant transformation (18).

### Screening

Transgender individuals face significant healthcare disparities and limited access to appropriate breast cancer screening; this contributes to delayed diagnosis and suboptimal care (27, 28). Progress has been made, however, to standardize screening practices for the gender-diverse patient population. The American College of Radiology published a comprehensive list of Appropriateness Criteria for transgender breast cancer screening in 2021 and an update of Appropriateness Criteria for cisgender female breast cancer screening in 2024 (29, 30). **Table 1** summarizes the organization’s recommendations, including which imaging modalities are “usually appropriate” or “may be appropriate,” depending on risk classification, age, gender identity, hormone therapy use, and if the patient underwent bilateral mastectomies. Annual screening breast imaging (including mammography and digital breast tomosynthesis) starting at age 40 is offered to all average-risk cisgender women, average-risk transgender women with at least five years of hormone use, and average-risk transgender men who have not undergone bilateral mastectomies.

**Table 1:** Breast cancer screening recommendations.

ACR screening recommendations (29, 30)	Cisgender women	Transgender women	Transgender men
	Average risk^*^	Average risk^*^ and ≥5 years hormone use^†^	Average risk^*^ without bilateral mastectomies^‡^
Usually appropriate	DBT/mammography screening	DBT/mammography screening	DBT/mammography screening
May be appropriate	US breast, MRI breast w/wo contrast		
	Intermediate risk^*^	Higher-than-average risk^§^ and ≥5 yrs hormone use^†^	Intermediate risk^‖^ without bilateral mastectomies^‡^
Usually appropriate	DBT/mammography screening	DBT/mammography screening	DBT/mammography screening
May be appropriate	US breast, MRI breast w/wo contrast, mammography with contrast		US breast, MRI breast w/wo contrast
	High risk^*^	Higher-than-average risk^§^ and no or <5 years hormone use^†^	High-risk^¶^ without bilateral mastectomies^‡^
Usually appropriate	DBT/mammography screening, MRI breast w/wo contrast	DBT/mammography screening	DBT/mammography screening, MRI breast w/wo contrast
May be appropriate	US breast, mammography with contrast		US breast

*Average risk is defined as < 15% lifetime risk, and screening is annually starting at age 40; intermediate risk is defines as 15-20% lifetime risk, and screening may start befor age 40; high risk is defined as >20% to 25% lifetime risk (including patients with a genetic predisposition to breast cancer, untested patients with first-degree relatives carrying a deleterious mutation, patients with history of thoracic radiation prior to age 30), and screening may start before age 40; patients with a personal history of high-risk breast lesions or breast cancer, dense breast tissue, or a family history of breast cancer may fit into intermediate-risk or high-risk.

^†^Screening is usually not appropriate for average-risk transgender women with no hormone use or <5 years of hormone use.

^‡^Screening is usually not appropriate for transgender men with bilateral mastectomies at any age for any risk.

^§^Higher-than-average risk includes patients with a personal history of breast cancer, chest irradiation before age 30, a genetic predisposition to breast cancer, a family history of breast or ovarian cancer, or untested patients with first-degree relatives with a genetic predisposition to breast cancer; screening starts at age 25-30.

^‖^Intermediate risk includes transgender men with a personal history of breast cancer, lobular neoplasiam atypical ductal hyperplasia, or 15-20% lifetime risk; screening starts at age 30.

^¶^High risk includes transgender men with a genetic predisposition to cancer, untested patients with a first-degree relative with a genetic predisposition to breast cancer, patients with thoracic irradiation before age 30, or >20% lifetime risk; screening starts at age 20-30.

Digital breast tomosynthesis (DBT), ultrasonography (US), Magnetic resonance imaging (MRI).

### Surgical management

In the non-oncologic setting, the goal of gender-affirming mastectomies for transgender men is to achieve a flat, masculine chest contour through glandular excision, skin recontouring, and modification of the nipple-areolar complex (31, 32). Incorporating chest masculinization into the surgical management of cisgender men and transgender men with breast cancer is appropriate when desired by the patient and safe from an oncologic standpoint (33). The choice of mastectomy incision, flap, and closure technique depends on a patient’s anatomy and aesthetic expectation. The double incision with free nipple graft provides broad exposure for glandular excision and masculinized contouring, making it well-suited for moderate to large chests (33, 34). Periareolar incision techniques may be appropriate for smaller chests but may leave residual retroareolar tissue, potentially limiting oncologic adequacy (31, 32). The wide-base bipedicled (WIBB) flap, utilized in nipple-sparing mastectomy, incorporates a mastopexy design to improve contour and closure during oncologic and gender-affirming procedures (35). The Angel Wing technique, developed for aesthetic flat closure following mastectomy, extends the incision laterally and superiorly to excise excess tissue in an elliptical fashion, improving lateral chest wall contour and symmetry by eliminating dog-ears or lateral adiposity. This approach is especially useful in patients not pursuing breast mound reconstruction (36). Adjunctive procedures such as pectoral implants that enhance chest projection may further support masculinization, particularly in delayed reconstruction (37). The Goldilocks procedure, which utilizes deepithelialized local tissue for volume restoration, may benefit patients who decline prosthetics but still desire chest projection in a non-delayed fashion (38). The patient in our case study underwent flat closure following his mastectomies and is considering delayed masculinization surgery.

Following an oncologic mastectomy, cisgender women and transgender women may be candidates for implant-based reconstruction, using either a two-stage approach with placement of a tissue expander followed by an exchange for a permanent implant, or a single-stage, direct-to-implant approach (39). Alternatively, autologous reconstruction uses the patient’s own tissue—most commonly from the abdomen, back, or thighs—to recreate a natural breast mound. Combination techniques may integrate implants, fat grafting, or autologous reconstruction to enhance contour, symmetry, or volume.

### Radiation oncological management

The administration of radiation therapy with respect to target volumes and organs at risk are similar among patients, regardless of gender identity, and depend on a patient’s anatomy and cancer stage. A discussion regarding potential radiation related toxic effects and their management is important and includes potential treatment related complications regarding breast/chest wall reconstruction (40–42). The patient in this case report had a pathologic complete response in the recovered lymph nodes, including the biopsied lymph node. A recent randomized clinical trial showed that regional nodal irradiation did not improve the invasive breast cancer recurrence-free interval at 5 years for select patients with clinical T1-3 and clinical N1 disease who convert to ypN0 disease following neoadjuvant systemic therapy (43). This trial, however, specifically excluded those with cN2 and cN3 disease. This trial’s design was consistent with previous data showing a significant disease-free survival benefit and overall survival benefit conferred by post-mastectomy irradiation for patients with clinical stage III disease (but not clinical stage I or II disease) after achieving a pathologic complete response to neoadjuvant chemotherapy (44). Many stage III patients in this study harbored cN3 disease. Considering the patient’s high axillary burden at diagnosis (including cN3 involvement), that only 3 sentinel lymph nodes were recovered, and the patient’s preference, his radiation oncologist recommended treatment. He received left chest wall and regional nodal irradiation with inclusion of the internal mammary chain. He received intensity modulated proton beam irradiation, which achieved significant reductions in integral dose to normal tissues, including the heart, left anterior descending artery, lung, and contralateral chest wall, compared to a rival photon plan. While modern techniques (such as deep inspiration breath hold, prone positioning, intensity-modulated radiation therapy, and volumetric modulate arc therapy) ensure that photon therapy is very safe for the majority of patients (45–48), proton therapy typically results in further reductions in dose to normal tissues. For select patients, this dosimetric advantage may confer a clinically meaningful decrease in the risk of pulmonary or cardiac toxic effects. Patients most likely to benefit from proton therapy include those who have challenging anatomy, a history of previous radiation therapy to the breast/thorax, a pathogenic germline variant in a cancer susceptibility gene (in which case sparing of non-target tissue decreases the risk for second malignancy), and those who require left-sided or bilateral regional nodal treatment (49–55). Multiple trials investigating the safety of de-escalating radiation therapy in select patients who respond well to neoadjuvant systemic therapy are ongoing and will help guide treatment recommendations for future patients.

### Biological subtyping and medical management

Breast cancer is classified based on tumor gene expression and molecular characteristics, particularly the expression of ER, PR, and HER2 (3, 56, 57). Four main biological subtypes are used in clinical practice: Luminal A, Luminal B, HER2-enriched, and basal-like (which overlaps considerably with triple negative breast cancer). These subtypes carry prognostic value, predict therapy response, and guide systemic therapy recommendations. ER signaling, for example, is targeted with endocrine therapy (e.g., selective estrogen receptor modulators/degraders and aromatase inhibitors), while HER2 signaling is targeted with monoclonal antibodies and antibody-drug conjugates. Specific regimens of cytotoxic chemotherapy, immunotherapy, and a variety of small molecule inhibitors are also administered depending on cancer subtype (58–60).

Breast tissue normally expresses the AR, which binds primarily testosterone and 5α-dihydrotestosterone. While generally growth inhibitory to mammary epithelial cells, androgens are also precursors to estrogens, which in turn may drive the development of ER-positive breast cancer (61, 62). Androgens have also been associated with HER2 overexpression and the activation of the epidermal growth factor receptor; this explains how androgens may act to drive ER-negative breast cancer (63, 64). The AR is expressed in ~75% of all breast cancers but more commonly in ER-positive disease (80-90%), compared to ER-negative disease (30-50%), HER2-positive disease (50-60%, regardless of ER status), and triple negative breast cancer (20-40%) (2–6, 65). Higher levels of circulating androgens, either because of higher endogenous production or exogenous testosterone use, may amplify signaling crosstalk among the AR, ER, and HER2.

A retrospective review of pathology specimens from female-to-male transgender patients undergoing gender-affirming mastectomies investigated the impact of exogenous androgens on breast tissue (66). This study showed that androgen-exposed breast tissue revealed dense fibrotic stroma, lobular atrophy, thickened lobular basement membranes, and gynecomastoid changes. Longer duration of androgen exposure was associated with more pronounced changes. The study also showed that ER and AR expression were highest in patients with intermediate duration of androgen exposure. Two additional studies compared pathology specimens from gender-affirming chest-contouring surgery in patients who did and did not receive testosterone therapy (67, 68). Longer duration of testosterone was associated with higher degrees of lobular atrophy but not fibrous content, decreasing amounts of epithelium and stroma, and a higher incidence of cysts, fibroadenomas, pseudoangiomatous stromal hyperplasia, and papillomas. For a subset of transmasculine patients who had a portion of the nipple-areolar complex available for evaluation, these specimens were compared to those of cisgender women who underwent a total mastectomy (69). The presence of Toker cell hyperplasia was higher in transmasculine patients than in cisgender women, and in cases of Toker cell hyperplasia, the rate of gland formation was higher in transmasculine patients. Further, the majority (90%) of Toker cells were AR-positive. These data underscore the complexity of extrapolating AR-targeted therapy evidence from cisgender women to gender-diverse populations.

The impact that AR expression has on breast cancer progression and prognosis is variable, depending on the co-expression or absence of ER and HER2 (7–15). A clinical meta-analysis evaluating over 10,000 patients with breast cancer reported a subgroup analysis of those with either ER-positive, ER-negative, ER-negative and HER2-positive, or triple negative disease. AR expression conferred improved disease-free survival and overall survival in all patients with ER-positive breast cancer (9). The same study showed a similar positive association with AR expression and clinical outcomes in patients with triple negative breast cancer. AR expression, however, did not improve clinical outcomes in all patients with ER negative breast cancer and was associated with worse overall survival in patients with ER-negative, HER2-positive breast cancer. These trends have been supported by multiple preclinical and clinical studies (3, 7, 10–13, 65, 70, 71) and have informed efforts to target AR signaling in patients with breast cancer.

While androgen deprivation has been a long-standing treatment approach for other AR-positive cancers—notably prostate cancer—the AR remains an emerging target in the treatment of breast cancer (72, 73). A phase II clinical trial enrolled patients with AR-positive, ER/PR-negative breast cancer and reported that bicalutamide, a nonsteroidal antiandrogen, produced a 19% clinical benefit rate at 6 months (74). Another phase II trial evaluated the efficacy and safety of the second-generation antiandrogen enzalutamide in patients with AR-positive triple negative locally advanced or metastatic breast cancer. This study showed 25% and 33% clinical benefit rates at 16 weeks in the intent-to-treat population and evaluable subgroup, respectively (75). Enobosarm, a novel oral selective androgen receptor modulator (SARM), was recently shown in a randomized, phase 2 clinical trial to have anti-tumor activity in patients with previously treated AR-positive, ER-positive, HER2-negative locally advanced or metastatic breast cancer (76). These and other drugs that modulate AR signaling, including 17 α-hydroxylase/17,20 lyase (CYP17) inhibitors, exogenous androgens (dehydroepiandrosterone, 4-OH-testosterone), androgen synthesis inhibitors, and AR-specific antisense oligonucleotides, are currently being investigated in clinical trials (72, 77).

AR signaling may be particularly relevant in patients with high levels of circulating androgens. Whether data from clinical trials investigating AR modulation (which enroll almost exclusively cisgender women) can be generalized to cisgender men and transgender men should be confirmed. In cisgender men with ER-positive breast cancer, the AR is typically targeted albeit inadvertently. Because of the conversion of androstenediones to estradiol through aromatase, current guidelines support the administration of gonadotropin-releasing hormone agonists with aromatase inhibitors in cisgender men. Tamoxifen is usually recommended first line, however, because of good tolerability and lack of high-level prospective studies in this patient population (78, 79).

### Management of symptoms of hypogonadism and gender dysphoria

The positive benefits that gender-affirming hormone therapy (GAHT) provide transgender individuals are well established. GAHT has been shown to improve mental health and quality of life measures, alleviate gender dysphoria by facilitating appearance congruence, and improve body satisfaction and social functioning (80–83). Thus, the significance of withdrawing GAHT should not be taken lightly: the potential oncological risk of continued GAHT should be weighed against the profound impact that withdrawal of this treatment might have on the patient’s gender identity and mental health. Ultimately, the treatment approach should be determined through shared decision-making with the patient. In addition to a multi-disciplinary cancer treatment team, mental health support, including social workers, counselors, family members, and patient interest groups is essential.

Cessation of testosterone in transgender men may result in a decrease in masculinization (e.g., decreased muscle mass, body hair, and libido), a return of feminine features (e.g., fat redistribution to the gluteofemoral region and increased pitch in voice), and mood disturbances related to gender dysphoria. Transgender men who have undergone menopause may experience symptoms related to estrogen deficiency, including vasomotor symptoms, fatigue, arthralgias, cognitive dysfunction, and disturbances in mood, sleep, and sexual function. Hormonal therapies are generally contraindicated in breast cancer survivors. Testosterone is not approved by the U.S. Food and Drug Administration for use in cisgender men with breast cancer (Depo testosterone package insert), and the existing data do not support the use of testosterone in transgender men with breast cancer. Hormone replacement with estrogen and progesterone has been associated with increased risk for breast cancer recurrence in a randomized clinical trial (HABITS) and a meta-analysis, especially in those with ER-positive tumors and cannot be recommended (84, 85). There is a lack of data investigating the impact that GAHT has on breast cancer in the transgender population. Further investigation is needed to determine if resuming GAHT after a period of cancer-free survival or if continuing on a reduced dose of GAHT (provided there are no other contraindications to this treatment) is safe from an oncological standpoint in the transgender population.

Nonhormonal options to manage vasomotor symptoms include selective serotonin reuptake inhibitors, serotonin norepinephrine reuptake inhibitors, gabapentin, oxybutynin, clonidine, and fezolinetant (86, 87). These and other symptoms related to the withdrawal of sex hormones may also be managed with dietary supplements, mind-body techniques (e.g., cognitive behavioral therapy, hypnosis), and various lifestyle modifications, including exercise, weight loss, trigger avoidance, and yoga (88).

## Conclusions

AR-targeted therapy in breast cancer is nuanced. Appropriate selection of agents that either block or potentiate AR signaling likely depends on a patient’s cancer biological subtype, levels and balance of circulating estrogens and androgens, and previous or concurrent exposure to ER-directed endocrine therapy. Targeting the AR may represent an underutilized treatment strategy, particularly for patients with high levels of circulating androgens, and should be further investigated. Nonhormonal pharmacologic options, lifestyle modifications, and mind-body techniques may help manage symptoms related to hormone withdrawal, including gender dysphoria in the transgender population.

## Data Availability

The original contributions presented in the study are included in the article/supplementary material. Further inquiries can be directed to the corresponding author.
